# Evaluating changes in radiation treatment volumes from post-operative to same-day planning MRI in High-grade gliomas

**DOI:** 10.1186/1748-717X-7-220

**Published:** 2012-12-21

**Authors:** Colin E Champ, Joshua Siglin, Mark V Mishra, Xinglei Shen, Maria Werner-Wasik, David W Andrews, Sonal U Mayekar, Haisong Liu, Wenyin Shi

**Affiliations:** 1 Department of Radiation Oncology, Kimmel Cancer Center and Jefferson Medical College of Thomas Jefferson University, 111 S. 11th Street, Philadelphia, PA, 19107, USA; 2 Neurological Surgery, Kimmel Cancer Center and Jefferson Medical College of Thomas Jefferson University, Philadelphia, PA, USA

**Keywords:** High-grade glioma, Radiotherapy planning, Same-day MRI, Glioblastoma multiforme, Anaplastic astrocytoma

## Abstract

**Background:**

Adjuvant radiation therapy (RT) with temozolomide (TMZ) is standard of care for high grade gliomas (HGG) patients. RT is commonly started 3 to 5 weeks after surgery. The deformation of the tumor bed and brain from surgery to RT is poorly studied. This study examined the magnitude of volume change in the postoperative tumor bed and the potential impact of RT planning.

**Method and materials:**

This study includes 24 patients with HGG who underwent craniotomy and adjuvant RT with TMZ at our institution. All patients had immediate postoperative MRI and repeat MRI during the day of RT simulation. Gross tumor volumes (GTV), clinical target volumes (CTV) of initial 46 Gy (CTV1) and boost to 60 Gy (CTV2) were contoured on both sets of MRIs according to RTOG (Radiation Therapy Oncology Group) guidelines. For patients who recurred after RT, the recurrence pattern was evaluated.

**Results:**

An average of 17 days elapsed between immediate and delayed MRIs. GTV1 (FLAIR abnormality and tumor bed) decreased significantly on the delayed MRI as compared to immediate post-operative MRI (mean = 30.96cc, p = 0.0005), while GTV2 (contrast-enhanced T1 abnormality and tumor bed) underwent a non-significant increase (mean = 6.82cc, p = 0.07). Such changes lead to significant decrease of CTV1 (mean decrease is 113.9cc, p<0.01), and significant increase of CTV2 (mean increase is 32.5cc, p=0.05). At a median follow-up of 13 months, 16 patients (67%) progressed, recurred, or died, with a progression-free survival time of 13.7 months. Twelve patients failed within all CTVs based on immediate and delayed MRIs, while one patient recurred outside of CTV2 based on immediate post-operative MRI, but within the CTV2 defined on delayed MRI.

**Conclusion:**

The postoperative tumor bed of HGGs undergoes substantial volumetric changes after surgery. Treatment planning based on delayed MRI significantly reduces the volume of treated brain tissue without local control detriment. The marked reduction of volume treated to 46 Gy based on delayed MRI scan, could result in increased sparing of organs at risk. There may be a small risk of inadequate radiation field design if radiation planning is based on immediate post-operative MRI.

## Background

High-grade gliomas (HGG), including World Health Organization (WHO) grade III anaplastic astrocytoma (AA) and grade IV glioblastoma multiforme (GBM), occur in approximately 5 cases per 100,000 people in the US. HGG are the most frequently occurring gliomas, with GBM comprising 53.7% of all new cases and AA accounting for 6.7% [1].

Maximum safe resection is standard initial treatment for HGG, and the extent of resection is correlated with improved outcomes [2]. Several studies have shown that post-operative radiation therapy (RT) alone or combined with chemotherapy improves survival in HGG [3,4]. RT was historically delivered via a whole brain approach. However, Shapiro *et al.* showed equivalence in survival in patients treated with whole brain RT to 43 Gray (Gy) with a cone-down to 60 Gy, resulting in a shift in treatment paradigm [5]. Further retrospective data from M.D. Anderson for HGG supported the efficacy of reduced field RT [6]. This approach is further supported by patterns of failure studies which, using combinations of biopsy, brain computed tomography (CT), brain magnetic resonance imaging (MRI), and autopsy information, have uniformly indicated that the predominant failure pattern for HGG patients is within a 2-cm margin of the tumor volume [7-9].

Given the use of reduced field, conformal RT, it is becoming increasingly important to accurately delineate the post-operative tumor bed. Recent standards for RT for HGG as denoted in Radiation Therapy Oncology Group (RTOG) 0825 guidelines recommend use of a CT-based simulation merged to a pre or post-operative MRI [10]. Guidelines on radiation target volume delineation remain unclear with some authors advocating for smaller margins than those described in the protocol [11,12]. In addition, guidelines on optimal timing of planning MRI are limited as well. The recommended starting time for radiation is 3-5 weeks (21–35 days) after surgery [10]. The brain deformation and change in the tumor bed during this time period is poorly understood. We hypothesized that large changes in the tumor bed and T2/FLAIR abnormality would occur between the time of surgery and initiation of RT. Therefore, a delayed MRI at the time of RT planning may more accurately reflect the tumor bed at time of RT, and potentially allow us to spare more normal tissue with no decrease in local tumor control. The purpose of this study was to prospectively evaluate the changes in treatment volumes between immediate post-operative MRI and same-day MRI at time of RT planning in an attempt to spare more normal tissue.

## Methods

### Study population

At our institution, all patients with HGG who undergo surgical resection and biopsy have immediate postoperative MRI (within 48 hours) to assess the extent of resection. We prospectively obtained a delayed MRI, which was performed at the time of RT planning, on 24 consecutive patients from July 19, 2010 to June 13, 2011, with pathologically confirmed HGG treated at our institution in an Institutional Review Board (IRB) approved protocol. All patients underwent craniotomy with resection or biopsy and were treated with temozolomide during RT and after. All data and medical records were reviewed comprehensively in accordance with the Health Insurance Portability and Accountability Act, our institutional IRB, and with the Helsinki Declaration of 1975, as revised in 2000.

### Contoured tumor volumes and treatment

Patients were simulated in the supine position with an aquaplast head mask. CT scan without intravenous contrast was obtained on a GE LightSpeed CT simulator (GE Healthcare, Chalfont St. Giles, UK) and 2.5-mm slices were obtained. Immediate post-operative MRI was obtained within 48 hours after craniotomy. Same-day MRI was obtained on the day of treatment planning. All patients underwent thin-cut, 1-1.5 mm slice thickness MRI imaging, including pre- and post-contrast T1-weighted and T2/FLAIR sequences. All images sets were imported into Elekta Focal Treatment Planning Software (Version 4.3.1, Stockholm, Sweden) and fused.

Target volume delineation was based on planning CT fusion with immediate and delayed MRI images. This provided us with fused images with high spatial fidelity (CT) and anatomic resolution (all MRI pulse sequences) upon which we could base target volume paired comparisons as well as design optimal treatment plans. The initial volume received a dose of 46 Gy. The initial gross tumor volume (GTV1) was defined as tumor bed and T2/FLAIR abnormality. Clinical tumor volume 1 (CTV1) was GTV1 with a 2-cm expansion, modified to avoid barriers of spread (i.e. bone, falx cerebri, ventricles). Planning tumor volume 1 (PTV1) was a CTV1 with a 0.5-cm uniform expansion. GTV2, which is the boost volume to 60 Gy, was defined as tumor bed and T1 post-contrast enhancement. CTV2 was a 2-cm uniform expansion of GTV2, modified to avoid barriers of spread. PTV2 was a 0.5-cm uniform expansion of CTV2. All the contours were generated on both immediate and delayed MRIs. All contours were done by a single operator (C.C.) and confirmed by two expert observers (W.S. and J.S.) in order to limit inter-observer variability. Both the operator and observers were blinded to whether the MRIs were post-operative or delayed and the MRIs were presented in a random manner to reduce recall bias.

### Power calculation

After retrospectively reviewing and calculating mean volume changes in 5 patients treated at our institution, and based on an estimated standard deviation of 25%, we required 24 patients to detect a difference of 30% in the CTV treatment volume with a two-sided hypothesis test at the 0.05 level of significance. These values were calculated at initial study design.

### Data analysis

Current RT treatment guidelines for HGG in RTOG protocols and many US institutions depend largely on T2/FLAIR and T1 post-contrast enhancement which led us to analyze target changes over time defined by these two standard pulse sequences. Treatment margins are based solely on these MRI findings and we set out to elucidate any changes between scans occurring in the immediate post-operative period and those at the time of treatment planning, often occurring 2-4 weeks after craniotomy. Student’s paired *t*-test was performed to compare the post-operative and delayed treatment volumes.

Recurrence patterns at interim analysis were then compared by fusion of the recurrence MRI with both treatment plans and all corresponding images. Recurrence was assessed individually on surveillance MRI. The site of recurrence was contoured on planning software and then compared with each blinded treatment MRI. Progression-free survival time was calculated using the Kaplan-Meier (KM) method. Location of recurrence within GTV1/2 and CTV1/2 based on post-operative MRI were compared to these volumes based on same-day MRI. Recurrence was based primarily on surveillance MRIs, which are reviewed at our weekly multidisciplinary brain tumor conference involving members from several institutional departments, including radiation oncology, neuro-oncology, neurosurgery, and neuroradiology. Biopsy-proven recurrence is not required in our institution if it is felt that imaging results show unequivocal progression by consensus of the board. Student’s paired *t*-test was performed to compare recurrence patterns between post-operative and delayed treatment volumes.

Statistical analyses were performed using the Stata Statistical package (version IC 11.1, Texas). Logistic regression was performed to identify patient and tumor variables predicting for a >30% change in CTV between the post-operative MRI and delayed MRI for both T1 and T2/FLAIR imaging sequences. Variables included in the analysis were steroid usage (dexamethasone), tumor histology, and extent of resection (gross total, subtotal, and biopsy). P-value of less than 0.05 was considered statistically significant.

## Results

In total, 16 patients with GBM and 8 with AA were included in the analysis. The median age at the initiation of RT was 57 years old (range 22-78). Please refer to Table 1 for patient characteristics. Treatment-planning CT scans of the brain were obtained a median of 17 days after craniotomy (range 7-32) and merged with immediate and delayed MRI for planning (see Table 2). While our institution generally prefers treatment planning 2 weeks after craniotomy in an effort to initiate treatment during postoperative week 3, this was not always possible due to extenuating patient circumstances. 

**Table 1:** Patient demographics

**Patient**	**Age**	**Histology**	**Location**	**Surgery**	*Dexamethasone treatment
1	52	AA	(R) Parietal	STR	No
2	56	AA	(L) Parietal	GTR	Yes
3	58	AA	(L) Temporal	STR	No
4	64	AA	(L) Temporal	STR	No
5	70	AA	(R) Temporal	STR	Yes
6	22	AA	(R) Frontal	STR	Yes
7	36	AA	(L) Frontal	GTR	Yes
8	59	AA	(L) Frontal	GTR	No
9	56	GBM	(L) Parietal	STR	No
10	78	GBM	(R) Parietal	STR	No
11	61	GBM	(L) Temporal	STR	Yes
12	59	GBM	(L) Parietal	STR	Yes
13	70	GBM	(R) Temporal	BX	Yes
14	48	GBM	(L) Frontal	STR	No
15	49	GBM	(R) Frontal	STR	Yes
16	66	GBM	(L) Temporal	STR	No
17	66	GBM	(R) Temporal	STR	Yes
18	50	GBM	(R) Frontal	GTR	No
19	46	GBM	(L) Temporal	STR	Yes
20	87	GBM	(R) Frontal	STR	No
21	35	GBM	(R) Thalamus	STR	No
22	75	GBM	(L) Frontal	STR	Yes
23	54	GBM	(R) Frontal	STR	Yes
24	52	GBM	(L) Frontal	STR	No

Abbreviations: *AA* Anaplastic astrocytoma, *GBM* Glioblastoma multiforme, *STR* Subtotal resection, *GTR* Gross total resection, *BX* Biopsy, *L* Left, *R* Right, *At time of MRI.

**Table 2:** Treatment volumes

**Patient**	**MRI Span (Days)**	**GTV1** Δ	**%**	**GTV2** Δ	**%**	**CTV1** Δ	**%**	**CTV2** Δ	**%**
1	8	-40.7	42	6.9	37	-139.4	26	37.9	16
2	10	-0.3	1	5.2	35	-13	5	18.2	8
3	7	-16.4	35	3	26	-77	20	16	8
4	15	-3.9	2	6.6	67	-49.9	5	47.6	26
5	19	-19.7	14	13.9	25	-49.4	7	33.9	7
6	26	-25.9	37	-17	37	-125.1	26	-89.8	24
7	17	-10.1	7	-5	4	-93.1	12	-35.8	6
8	13	-2.4	36	-0.3	14	-27.5	17	-3.4	3
9	13	-12.4	27	3.3	44	-83.1	20	15.1	22
10	27	-21.8	33	1.9	31	-154.6	23	18	12
11	17	-52.7	34	22	39	-195.9	24	198.8	83
12	11	-12.4	18	2.2	7	-69.4	14	1.4	0
13	15	-14.3	16	22.6	37	-16.9	3	136.9	31
14	16	36.9	60	16.2	107	184.9	24	85.8	37
15	12	-35.8	31	-10.8	27	-95.8	18	-43	12
16	32	-72	63	-0.2	1	-282.8	42	45.2	16
17	14	-130.9	54	14.6	20	-347.7	34	64.4	12
18	28	-35.5	44	1.8	6	-160.3	31	-14.6	5
19	16	11.6	100	-13.4	54	36.7	13	-5.1	3
20	19	-140.1	56	6.3	19	-300.6	28	31	9
21	31	-17	13	-10.6	9	-101.1	14	-82.2	12
22	22	-2	3	-2	21	-10.1	2	-10.5	6
23	14	-5.2	3	73.3	121	-27	3	249.4	51
24	16	-46.3	26	19.4	36	-164.6	19	65.4	16
Average	17	-31	22	6.8	22	-113.9	17	32.5	12

Abbreviations: *Post-op* Post-operative, *MRI* Magnetic resonance imaging, *GTV1* Gross tumor volume treated to 46 Gy, *CTV1* Clinical tumor volume treated to 46 Gy, *GTV2* Gross tumor volume treated to 60 Gy, *CTV2* Clinical tumor volume treated to 60 Gy, (-) values indicate volume was smaller on same-day MRI, (+) values indicate volume was larger on delayed MRI.

### Volumetric analysis

There were significant changes in both T1 post-contrast enhancement and T2/FLAIR abnormality from immediate post-operative MRI to delayed MRI scans in all patients. T2/FLAIR-based GTV1 decreased in 23 patients, with an average decrease of 22.1% (mean = 30.96cc, range -140.15 to -0.33, p = 0.0005). CTV1 had a 16.9% average decrease in volume (mean = 113.86cc, range -347.70 to 36.73, p = 0.000003).

**Figure 1:**
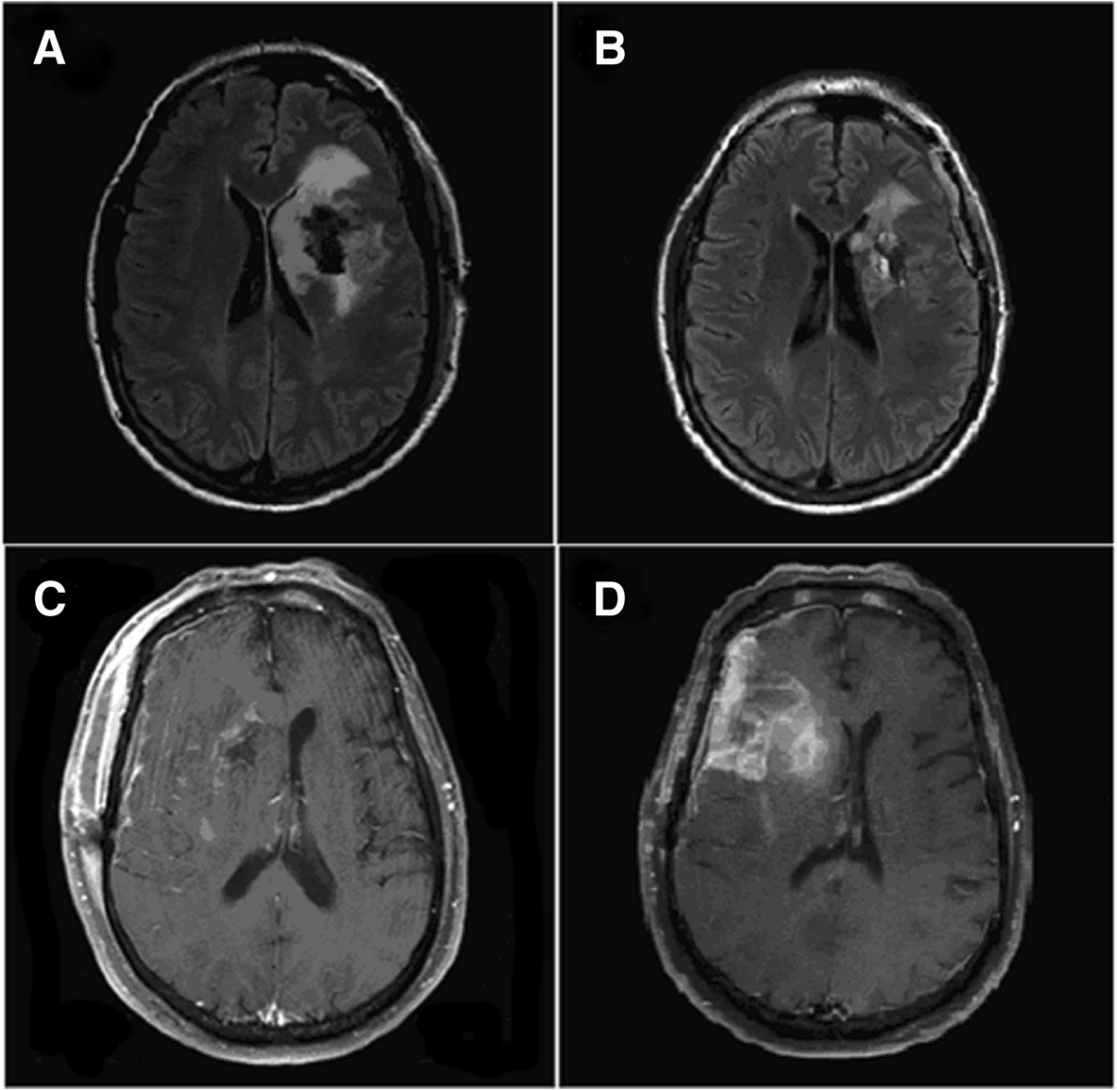
**Changes in volume on T2/FLAIR and T1 contrast-enhanced MRI: A. T2/FLAIR MRI performed 1 day after craniotomy. B**. T2/FLAIR MRI performed 2 weeks after craniotomy. Another patient with **C**. T1 contrast-enhanced MRI performed 1 day after craniotomy. **D**. T1 contrast-enhanced MRI performed 2 weeks after craniotomy.

**Figure 2:**
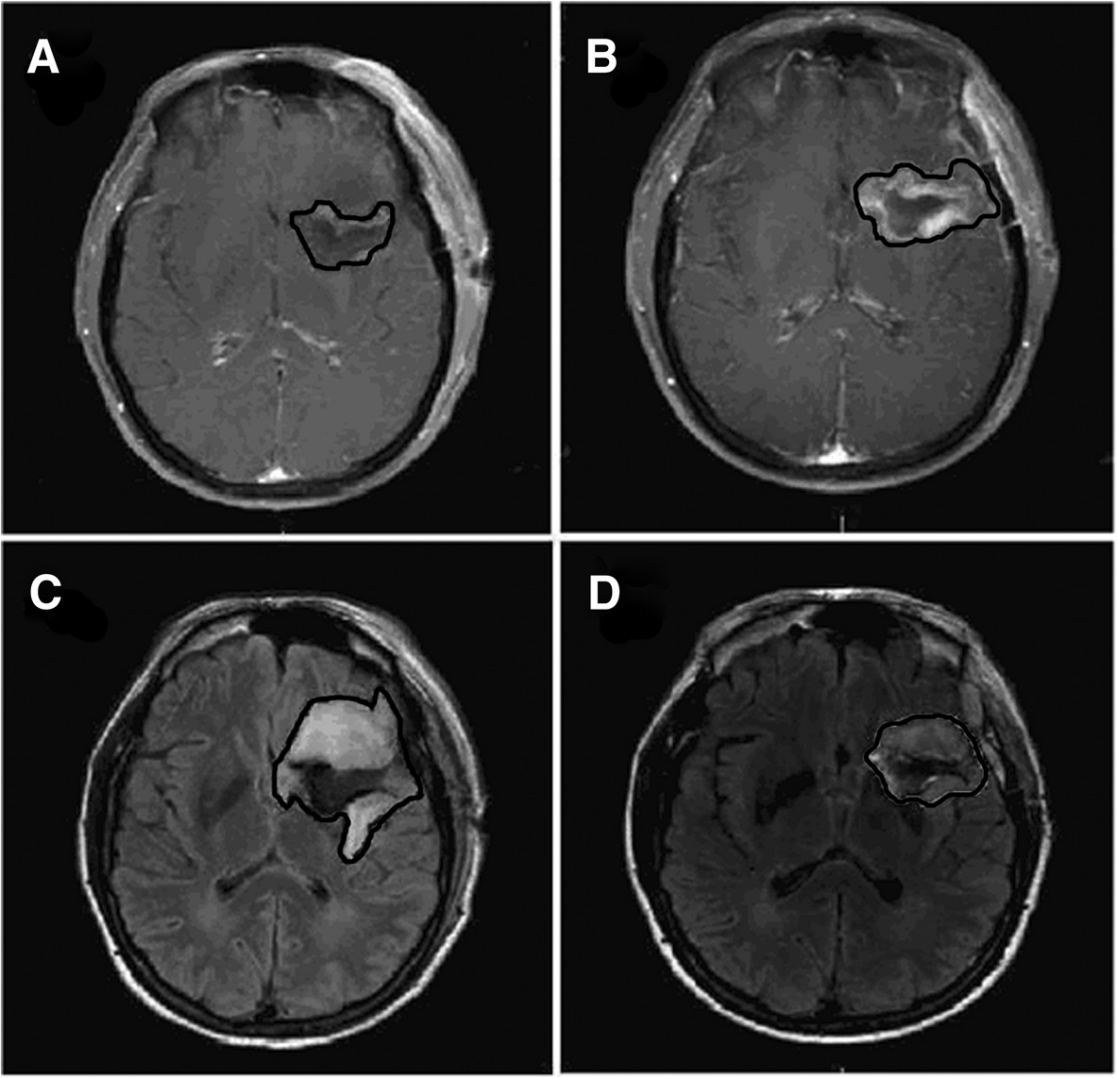
**Changes of GTV on T2/FLAIR and contrast-enhanced MRI: A. GTV2 on post-operative T1 contrast-enhanced MRI. B**. GTV2 on delayed T1 contrast-enhanced MRI, **C**. GTV1 on post-operative T2/FLAIR MRI. **D**. GTV1 on delayed T2/FLAIR MRI.

T1 post-contrast enhancement-based GTV2 underwent an average increase of 22% (mean = 6.82cc, range -13.44 to 73.26, p = 0.07). This resulted in a 12% increase of CTV2 (mean = 32.52cc, range -89.83 to 249.45, p = 0.05). Thirteen patients in total had a 30% or greater increase in GTV1 (n= 9/16 GBM patients), while 11 patients had a 20% or greater increase in CTV1 (n=8/16 GBM patients, n= 3/8 AA patients). Several lesions had a striking change in size, including a CTV2 increase of 83% (Figure 1C and D), a CTV2 increase of 51% (Figure 2), and a CTV1 decrease of 42%; all were GBMs. 

In subgroup analysis, AA GTV1 volume underwent a 16% decrease (mean = 14.94cc, range 0.33 to 40.73, p = 0.02). CTV1 decreased by 13% (mean = 71.81cc, range -12.96 to -139.43, p = 0.002). However, changes in GTV2 (5%, mean = 1.67cc, range 0.30 to 16.96, p = 0.63) and CTV2 (1%, mean = 3.05cc, range -89.83 to 47.60, p = 0.86) were non-significant.

In contrast, GTV1 for GBM patients underwent broad fluctuations in the post-operative and delayed GTV volumes, with a decrease in volume of 32.82% (mean = 38.97cc, range 23.19 to 189.32, p = 0.003). Similarly in significance, CTV1 decreased by an average of 20.01% (mean = 134.88cc, range -300.64 to 36.73, p = 0.0008). GTV2 underwent lesser changes that trended toward significance, with an increase of 23.44% (mean = 9.40cc, range -13.44 to 73.26, p = 0.09). CTV2 reached statistical significance with an increase in size by 14.43% (mean = 47.25cc, range -82.18 to 249.50, p = 0.05).

When comparing volume changes with steroid usage, tumor histology, and type of resection, no significant effects in treatment volume were appreciated when logistic regression was performed.

### Recurrence patterns

**Figure 3:**
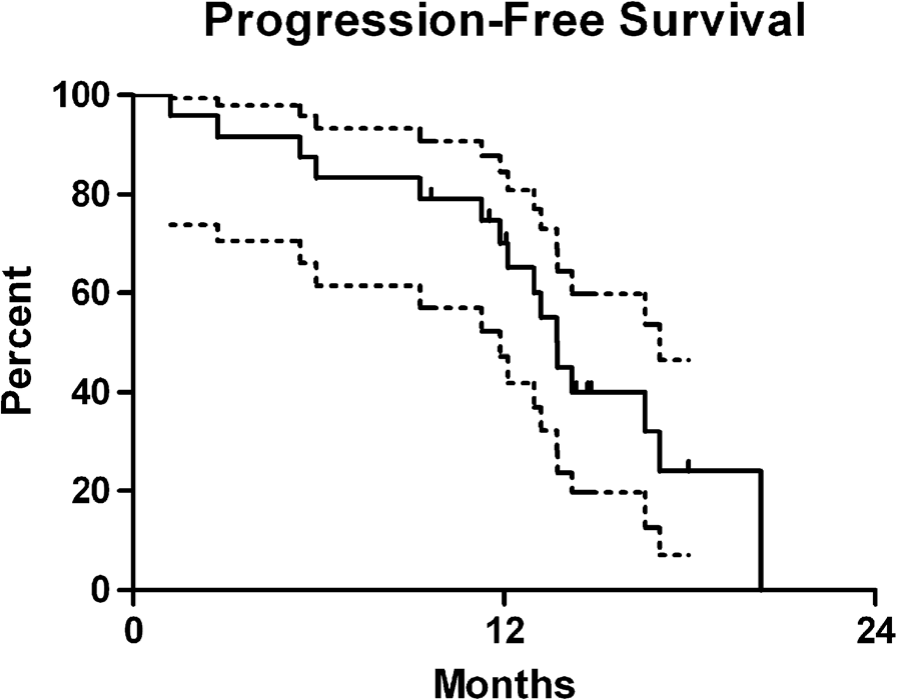
Progression-free survival.

At a median follow-up of 13 months, 16 patients progressed or died. One patient recurred distantly and 2 died with no sign of progression. Progression-free survival time was 13.7 months, on KM analysis (Figure 3). Failure was within CTV1 and CTV2 based on both immediate and delayed imaging in 86% of patients (n=12/14). One patient failed outside of CTV2 based on immediate post-operative MRI only and no patients failed outside of CTV1 or CTV2 based on same-day imaging. This was not statistically significant (p=0.34). Though not significant, 46% of patients (n=6) failed outside of one or more GTV volumes on post-operative MRI and 39% of patients (n=5) failed outside of one or more GTV on same-day MRI (p=0.35). 

## Discussion

Long term survival of patients with HGG remains poor due to local recurrence after treatment, with the vast majority occurring within a 2-cm area of the presurgical tumor margin, emphasizing the importance of accurately defined treatment volumes [7-9]. Guidelines for treatment planning have been evolving in recent RTOG protocols, which are used as guidelines for treatment planning by many institutions within the US and internationally. While the recent RTOG 0825 protocol required CT-based planning for RT with post-operative MRI fusion to ensure accurate target delineation, only three years earlier RTOG 0525 used pretreatment MRIs for treatment planning [10]. While CT often displays the surgical tumor bed, MRI presents the planning physician with further tools to elucidate tumor bed and surrounding edema [13]. Contrast-enhanced T1 MRI best evaluates residual tumor and areas of blood–brain barrier breakdown, while T2/FLAIR enhancement best evaluates tissue edema [14,15].

Our data shows that the CTV1, including the resection cavity and surrounding edema, changes extensively in both GBM and AA patients in the small time interval between surgery and RT planning, with same-day planning MRI elucidating a more accurate representation of the lesion and decrease in surgical edema. CTV2 also changed significantly for combined AA and GBM, but only for GBM patients on subset analysis. Twenty-three of 24 CTV1 volumes decreased in size within an average of 17 days between post-operative MRI and treatment planning MRI. This decrease in size can potentially spare proximal organs at risk and spares normal brain tissue from being treated to the initial dose of 46 Gy. In contrast, CTV2 increased in size in 16 of 24 patients, which could potentially result in improved tumor bed coverage during the treatment boost in comparison to immediate post-operative MRI. Nearly a third of all patients had over a 25% change in CTV2 with planning MRI performed in 17 days or less after craniotomy, including 2 patients with a greater than 50% change in their volumes.

Our data also shows that treating with RT based on same-day MRI does not result in increased rates of tumor recurrence outside of the treatment volume, as no failures were seen outside of the CTV volumes based on same-day MRI. In fact, only one patient had disease recurrence locally which was outside of CTV based on post-operative imaging only. Therefore, while less volume of tissue is irradiated when planning is based on same-day MRI versus immediate post-operative MRI, it is unlikely that treatment based on same-day MRI results in an increase in tumor-miss. These changes could result in alterations in treatment volumes, resulting in a large impact for future RTOG and other protocols, which may be based on imaging sets that do not accurately define the tumor bed volume.

Similar findings regarding volume changes were presented at the 2010 American Society for Radiation Oncology Annual Meeting, revealing large variations in surgical cavities with both expansion and shrinkage occurring mostly within the first two weeks after craniotomy for HGG patients. This group of investigators also demonstrated a benefit in local control and a decrease in radiation injury in patients with planning MRI delayed for over two weeks [16]. While we cannot determine a benefit in local control as the aggressive nature of these tumors results in frequent local recurrence, it appears that when MRI is delayed over two weeks, normal tissue can be spared with no detriment on local control.

While RTOG 0825 has a clear set of guidelines concerning treatment margins, some practitioners advocate tighter margins [11,12]. However, when reviewing our data, caution should be employed with this technique as it introduces the potential of missing gross tumor, as our data indicates that GTV increases even in the short time period between post-operative and same-day MRI, timing of MRI may become more important for practitioners of limited-margin treatment to limit the possibility of tumor miss.

While we have shown that the resection cavity undergoes extensive volumetric changes in the short interval between surgical resection and RT planning, several studies have illustrated volumetric changes throughout treatment. Shukla *et al.* have shown that at week 5 of treatment, 12/15 patients with unifocal disease had a median reduction of 54.85cc in tumor volumes on T2-weighted MRI [17]. As a result, they suggest mid-treatment re-planning MRI, potentially to define boost fields. Tsien *et al.* prospectively followed 21 patients who were rescanned at week 3 during RT [18]. They found an increase in GTV in 3/12 patients with GBM, with all cases requiring increase in size of treatment fields. A multi-institutional trial examining volume changes midway through treatment of GBMs showed a change in GTV in 80% of cases [19]. Given the increasing impetus to reduce treatment volumes recently, further research in this area may be warranted.

It is possible that the treatment volume undergoes several changes as the resection space contracts, surrounding edema waxes and wanes, and the actual tumor volume changes, and no minimal amount of reasonable reimaging could monitor this change. While the decrease in treatment volume seen on T2/FLAIR imaging in our study was likely from a reduction in peri-tumor edema, the increase in GTV2 was somewhat unexpected. This change may represent evolution of blood products, principally methemoglobin, post-operative changes which have been documented to occur between days 5 and 14 post-operatively, and/or tumor progression [20]. Optimal imaging times remain unknown but target volumes may be least ambiguous after 14 days postoperatively. As a result, we recommend that the MRI used to define the planning volume should be delayed to the time of CT simulation, with the treatment to start as soon as possible to reduce the potential for further changes that might negatively impact the effectiveness of treatment.

## Conclusions

The results of this study illustrate the changes that the post-operative tumor bed of high-grade gliomas undergoes prior to RT and the important effect on target delineation during treatment planning. The marked reduction of volume treated to 46 Gy based on same-day versus post-operative MRI may result in better sparing of normal brain tissue without a detriment in local control. Additionally, the increased CTV2 volume may result in better tumor coverage, avoiding potential geographic miss in treatment based on immediate post-operative MRI scan. Given the differences demonstrated in treatment volume at varying MRI time points, further attempts should be made to correlate treatment planning with patterns of failure to maximize local control while minimizing toxicity.

## Competing interests

The authors declare that they have no competing interests.

## Authors’ contributions

CC and WS designed the study, performed radiation planning, analyzed data, and drafted the manuscript. JS, MM, SM helped perform radiation planning, analyzed data, and helped draft the manuscript. MW, DA, XS, HL helped analyze data and draft the manuscript. All authors read and approved the final manuscript.

## Funding support

Research was supported in part by the Kimmel Cancer Center’s NCI Cancer Center Support Grant P30 CA56036.
